# Performance of the SABAT Neutron-Based Explosives Detector Integrated with an Unmanned Ground Vehicle: A Simulation Study

**DOI:** 10.3390/s22249996

**Published:** 2022-12-19

**Authors:** Michał Silarski, Marek Nowakowski

**Affiliations:** 1 Faculty of Physics, Astronomy and Applied Computer Science, Jagiellonian University, 30-348 Cracow, Poland; 2 Military Institute of Armoured and Automotive Technology, Okuniewska 1, 05-070 Sulejowek, Poland

**Keywords:** neutron activation analysis, γ spectroscopy, non-destructive sensors, mobile robot

## Abstract

The effective and safe detection of illicit materials, explosives in particular, is currently of growing importance taking into account the geopolitical situation and increasing risk of a terrorist attack. The commonly used methods of detection are based predominantly on metal detectors and georadars, which show only the shapes of the possible dangerous objects and do not allow for exact identification and risk assessment. A supplementary or even alternative method may be based on neutron activation analysis, which provides the possibility of a stoichiometric analysis of the suspected object and its non-invasive identification. One such sensor is developed by the SABAT collaboration, with its primary application being underwater threat detection. In this article, we present performance studies of this sensor, integrated with a mobile robot, in terms of the minimal detectable quantity of commonly used explosives in different environmental conditions. The paper describes the functionality of the used platform considering electronics, sensors, onboard computing power, and communication system to carry out manual operation and remote control. Robotics solutions based on modularized structures allow the extension of sensors and effectors that can significantly improve the safety of personnel as well as work efficiency, productivity, and flexibility.

## 1. Introduction

The growing scale of the devastation which can be caused by even a single terrorist attack requires more effective methods for the detection of explosives and other hazardous materials (e.g., chemical agents). The limitations of commonly used methods and the growing need for mobile devices, allowing for effective and rapid recognition, has led to a constant search for novel solutions.

Currently, the state-of-the-art methods of detecting hazardous substances are based primarily on the use of X-rays, which interact with electrons and thus provide determination of the density distribution and the shapes of tested subjects but do not allow for exact identification. In an aquatic environment, for the detection of war remnants and dangerous chemicals, one uses primarily sonars, which allow one to determine only the position and shape of the object without giving information about its chemical composition. Therefore, the detection of any suspicious object requires additional verification. The disadvantages of the above-mentioned methods are not present in devices based on a stoichiometry analysis by irradiating the substance with neutrons and measuring the energy spectrum of emitted γ quanta. Most of the illicit substances are composed of oxygen, carbon, hydrogen, and nitrogen. Chemical agents contain also sulfur, chlorine, phosphorus, and fluorine. In addition, their elemental composition is different from the composition of most of the materials commonly used in industry and in everyday life. Thus, these substances can be unambiguously identified by the determination of the ratio between the number of C, H, N, O, S, P, and F atoms in a molecule, which can be achieved noninvasively by applying neutron activation analysis (NAA) techniques [1].

**Figure 1:**
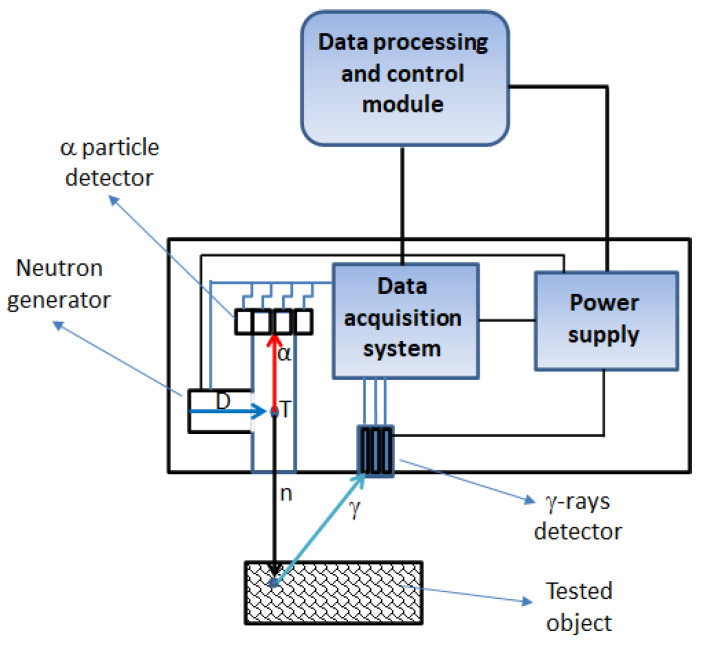
Scheme of the neutron-based sensor developed within the SABAT project. Neutrons are generated through deuterium–tritium (DT) collisions, which also result in α particle creation. Signals from both the γ-rays and α particles are transferred to the data acquisition system, which measures their charges and times of arrival. Events with coincident registration of both particles are then transferred to the data-processing module. Moreover, this mode of operation significantly reduces the environmental background.

In the world, there are several already-developed devices based on neutron activation (see, for example, [2,3,4]), but their mobility is limited, and the irradiation time for the stoichiometry recognition may be on the order of several minutes, especially if the object is covered or buried. Thus, neutron-activation-based sensors are still under intense development. One such device has been developed at the Jagiellonian University in Kraków within the SABAT project devoted to the detection of munitions, chemical agents, and heavy fuel oil sunk in the Baltic Sea [5,6,7,8]. A schematic view of the SABAT sensor is presented in Figure 1. The suspected item is irradiated with a flux of neutrons produced using a compact deuterium–tritium (DT) generator. As a result of the DT nuclear reaction, an alpha particle is created together with the neutron, which is emitted nearly isotropically, with a well-defined energy equal to about 14.1 MeV [9]. Neutrons are absorbed or scattered inelastically on nuclei of the investigated object exciting them. The activated nuclei de-excite to the ground state, emitting γ quanta whose energies are characteristic for each isotope [5]. These quanta can be detected by a scintillator or semiconductor detector, which enables the reconstruction of the elemental content of the tested substance and, as a consequence, its identification [6]. 

Considerable background in this type of measurement arises due to the registration of γ quanta from neutron interactions with the environment. Reduction of this noise is especially important in the underwater applications of neutron activation techniques due to the high attenuation of neutrons and their interactions with water resulting in background lines of oxygen and hydrogen. One of the methods of coping with this problem is the registration of the α particle, originating from the DT reaction, emitted in the opposite direction to the neutron (so-called associated particle imagining, API). However, it also decreases the effective neutron yield usable for the interrogation and additionally increases the time needed to decide if the inspected object is dangerous. In most cases, this additional system also significantly increases the weight of the neutron generator used as the neutron source. Therefore, for the ground applications, we assumed the use of lightweight and compact generators without the API modality. One such solution may be the Thermo Fisher Scientific P-320 source [10]. We propose using compact scintillating detectors based on LaBr_3_:Ce:Sr crystals [11] read out by a matrix of silicon photomultipliers (SiMPs), providing a determination of the position of the γ-ray hit [12]. We plan to use an active cover of the main scintillating crystal, which may further decrease the background and the time needed to gather sufficient data to detect the threat [13]. The γ-ray detector used in the SABAT sensor is characterized by a very good energy resolution (~3% at the 662 keV line) and acceptable timing properties (~500 ps time measurement resolution) [12]. As presented in Figure 1, signals from both the neutron generator and γ-rays detector are transferred to the data acquisition system (DAQ), which is able to register their times of arrival and charges. In the case of API application, γ-rays are detected in coincidence with signals from the α particle detectors, which provides reduction of the environmental background. Alternatively, in the case of the pulsed mode of neutron generator, one can register the time of the pulse generation and use it as a start signal for the γ-ray detector. This allows separation of the prompt γ quanta from neutron inelastic scattering from those generated in the capture processes and increases the performance of detection [5]. In the first prototype of the SABAT sensor, we use the CAEN A5202 unit based on two Citiroc-1A chips produced by WeeROC, providing 64-channel readout [14]. Each readout channel is composed of a preamplifier, a slow shaper with peak sensing detector and an ADC, and a fast shaper followed by a discriminator. The 64 channel self-triggers (discriminator outputs) can be used for counting, time stamping, time over threshold (ToT) measurement, and generation of the board bunch trigger. CAEN A5202 can be connected to a data-processing and control unit (e.g., mini-PC) via USB or Ethernet [14]. The dimensions of the main components of the sensor determine its dimensions and weight. The whole system can be confined within a 50 × 15 × 10 cm^3^ box, and its mass does not exceed 12 kg. Apart from compactness, our sensor is characterized by low power consumption (~70 W), which allows usage of batteries installed onboard a carrying platform, e.g., a drone. Apart from the possibility of non-invasive recognition of illicit materials another advantage of the SABAT (and other neutron-based solutions) is the response time, which is determined almost solely by the time of de-excitation of irradiated nuclei. This time does not exceed 100 μs and originates mainly from the neutron capture γ quanta delayed due to the thermalization of fast neutrons in the irradiated object. The response time of the γ-ray detector itself is dictated by the signal rise time (a property of the scintillator crystal used) and the transit times of the photomultipliers, which amount to tens of nanoseconds and are negligible with respect to the thermalization times of fast neutrons. In turn, the scintillator properties influence sensor recovery time depending predominantly on the decay time. In the case of LaBr_3_:Ce:Sr this quantity amounts to about 16 ns [11]. A potential radiation hazard due to the production of long-lived isotopes in the activation process is negligible due to relatively low neutron fluxes used during the inspection. However, our sensor must be operated remotely with a safety zone of 15–20 m to reduce possible exposure to unnecessary radiation doses.

The neutron-based detector can be used in various environments, such as non-invasive underwater detection of hazardous materials or defining hidden threads concealed under the ground as well as illicit materials explosives that can be found in public places such as airports. Each application has different requirements considering the location of the sensor, type of used platform (vehicle), mounting method, and required time for measurements. Exploring the underwater environment using unmanned underwater vehicles (UUV) should take into account disturbances (drag effects, ocean currents), and it is very difficult to keep the vehicle on the required path and complete the underwater mission. On the other hand, adoption of unmanned aerial vehicle (UAV) solutions in daily life scenarios, due to the advancement of components, allows one to autonomously conduct a variety of operations that can support the remote detection of hazardous materials. They are commonly used in applications such as inspecting pipelines and power lines or even detecting gas [15]. They can be used in environmental monitoring and threat detection even in relatively large areas, supporting 3D mapping [16]. Some such devices are based on nano-drones equipped with lightweight and compact biosensors capable of detecting real-time odorant concentration differences in air and of tracing odor sources [17,18]. Despite many advantages, drones are vulnerable to weather conditions and environments with high vegetation, which cause the potential risk of ground impact or damage, limiting their usage in some missions.

In our study, we have investigated many different operational conditions. Authors focused on a very real issues related to the presence and location of landmines in the surveilled area. Currently, a very important task is helping civilians in mine-contaminated areas to reduce risk by mapping and planning removal action. It should be stressed that explosives can remain hidden in wild above-ground vegetation, so authors pay attention to select an unmanned ground vehicle (UGV) that can carry out missions in unknown environments. The dimensions and weight of the analyzed neutron-based detector have influenced the adopted wheeled platform’s ability to precisely operate in suspected hazardous areas. It should be emphasized that the proposed solution, a remotely controlled platform with optional autonomous mode, provides fast and easy movement over rough terrain. Optimized SABAT sensors integrated into the mobile robot can also effectively perform detection of improvised explosive devices (IED), mines, or other threats, e.g., at airports

**Figure 2:**
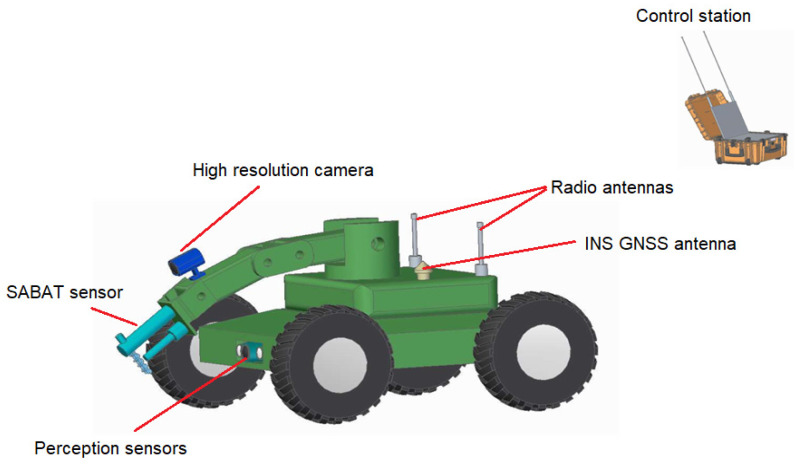
Schematic view of the SABAT sensor integrated with a ground vehicle simulated in this work. The sensor (light blue) is mounted on a manipulator arm with a camera (dark blue).

Such platforms are developed at the Military Institute of Armoured and Automotive Technology in Sulejówek, Poland. They provide modern obstacle detection systems and wireless data transmission. The integration of the SABAT sensor will be the first step towards an autonomous system for the detection of hazardous materials on the ground based on neutron activation techniques. In this article, we present the first series of Monte Carlo simulations that will be used to optimize the geometry of our sensor installed on the vehicle presented in Figure 2.

## 2. Materials and Methods

Described mobile robot in the form of the wheeled vehicle allows remote operation on the ground (unmanned ground vehicles). The platform is equipped with a real-time embedded system with sensors for environmental perception as well as peripheral elements to perform actions related to the detection of threats.

The adopted UGV consists of the following modules:

- The chassis with electric drives, batteries, and control panel and electric energy distribution circuits (Table 1);
- Sensors and cameras;
- IT infrastructure containing an on-board computer and radio communication transceivers;
- An arm to install the developed detector;
- Control station with the user application;

Operation in hazardous environments requires the orientation of the mobile robot in the space based on a GNSS signal (Global Navigation Satellite Systems, Inertial Labs, Inc., Paeonian Springs, VA, USA) or estimated position using an aided inertial navigation system (INS) combined with fitted external wheel speed sensors. UGV allows the creation of environmental map by transferring measured data (referring to the detected explosives) and indicates its location on this map during missions. Information about the world around mobile robots can be used for decision-making processes locally by operators or transferred to external management systems.

Mobile robots can move also using a vision and perception system without the use of navigation devices in an environment. The robot includes two high-resolution cameras; the main camera is mounted on the front side of the chassis, and a detail camera is attached at the end arm for a detailed view. The main camera has a night vision system to operate in darkness without the use of lights. Additionally, the platform is equipped with sophisticated sensors to detect objects around UGV to support operator tasks. High-resolution 3D and 2D data can navigate safely by recognizing and avoiding typical obstacles.

**Figure 3:**
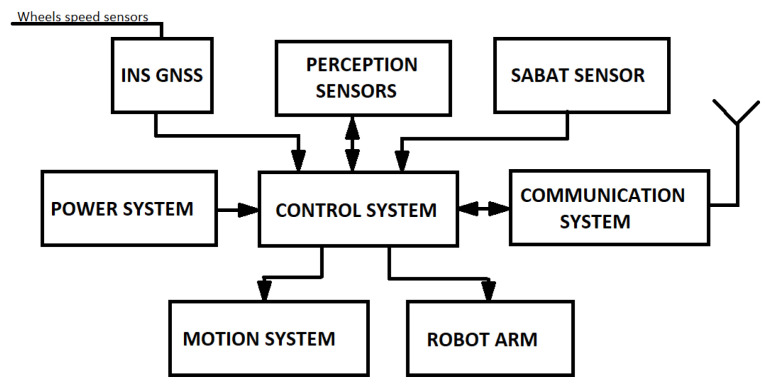
System architecture of the UGV which will be used to carry the neutron-based sensor.

The control system was designed to manage internal and external information, receives commands from the remote control station, and transmits measuring data and other parameters related to the unmanned platform to the operator. The architecture of the embedded control system is shown in Figure 3.

The proposed modular architecture allows the extension of additional detectors or sensors depending on user configuration. The main interface for data exchange is Ethernet. In order to ensure wireless communication, it is necessary to use a radio link based on modems, enabling the transmission of data and video streams from the vehicle to the operator system with minimal delays [19].

The main issue is securing data transfer in a real-time regime between the operator and mobile robot during field operations. It should be stressed that control of vehicles is based on installed sensors or using built-in autonomous functions. It must be taken into account that the useful operating range of the neutron-based detector is about 20 cm (see Section 3—Results) and requires constant communication with the UGV to ensure proper transmission of location data or video stream (teleoperation mode), especially while explosives have been already detected in the surrounding area. Defined potential detection zone of hazardous substances has an influence on scenarios of operation in an unknown environment in which typical short-range networks could be easily disturbed, causing loss of control. Additionally, the transmission of video consumes significant resources. Therefore, authors proposed the use of a wideband radio system, ensuring high stability (multipath routing) based on experience in similar projects of autonomous vehicles by the Military Institute of Armoured and Automotive Technology.

**Figure 4:**
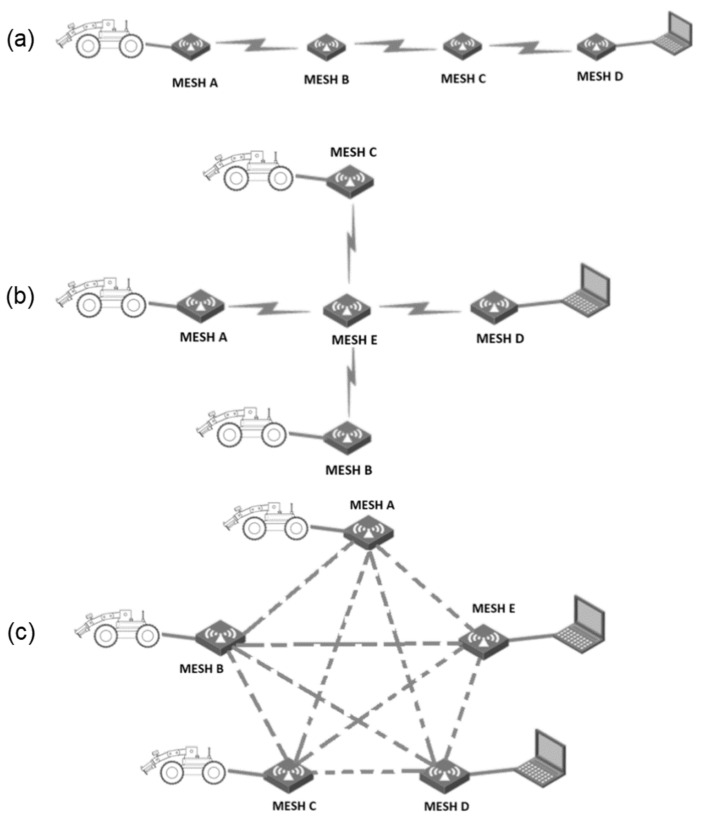
Operational modes of mesh topology: cascade connection (**a**), star topology (**b**), grid (**c**).

The selected modem is designed to create mobile multi-node MESH radio communication networks that allow information to travel from node to node without delays or failures. Each radio modem is a node of a self-configuring network that automatically becomes part of the network’s existing structure (see Figure 4). Such functionality allows the extension of the operational range and reduces potential interferences with other wireless devices. The datalink operates in the 1400–1450 MHz frequency band.

Performance studies of the neutron-activation-based SABAT sensor presented in this article concentrate on the determination of the basic practical characteristics assuming the simplest and cheapest solution which did not include any background suppressing techniques described in Section 1. To this end, we have performed Monte Carlo simulations with the Monte Carlo N-Particle Transport (MCNP) v6.11 [20] package, a general-purpose, three-dimensional simulation tool able to transport many particle types (including neutrons and γ-rays) in a broad range of energies in a realistic manner. It allows the determination of nuclear criticality, dosimetric quantities, and detector response and may be used for radiation shielding designs and many other applications. Transport of neutrons is performed using cross-sections from the Evaluated Nuclear Data Files (ENDF71x) library [21] down to the thermal energies. The latter ones are propagated according to free gas and S(α,β) models [20]. For the γ quanta, coherent and incoherent scattering is taken into account, the photoelectric effect with a possible fluorescent emission and pair creation processes. As was mentioned before, as a neutron source, we considered the newest version of the compact and lightweight DT generator by Thermo Fisher Scientific, P-320 [10], providing 10^8^ neutrons per second. As in the previous studies, the assumed γ quanta spectrometer is a scintillator detector with a 2″ × 2″ LaBr_3_:Ce:Sr crystal read out by a photomultiplier tube [5]. The scenario simulated in this work contains a realistic model of the generator and detector installed on the vehicle described at the end of Section 1 operating at different distances from the tested object placed on the ground or buried in the soil at various depths. The virtual scene used in the simulations was constructed as a box with dimensions 450 × 450 × 400 cm^3^ filled with air and a 200 cm layer of soil. This ensured that the environmental background, which will be present in the real measurements, is modeled in a realistic way. As the illicit material, we have simulated a box of TNT with dimensions 22 × 32 × 8.6 cm^3^ and mass of about 13 kg, corresponding to an anti-tank mine MPP-B [22] with a composite cover of a few millimeters. The neutron generator dimensions were taken from [10], and the simulated point source emitting 14 MeV neutrons was placed in a position corresponding to the tritium target of the real generator. The simulated generator tube was made from steel and filled with vacuum. Steel was assumed also as the main material building the UGV platform with realistic dimensions (105 × 85.6 × 39 cm^3^). Materials compositions, implemented according to the atomic fractions, were taken from the commonly available PNNL-15870 rev. 1 library [23]. The simulations were performed in view of the γ quanta detector response and identification of lines corresponding to the elemental composition of TNT: 2.23 MeV for hydrogen, 4.44 MeV for carbon, 6.13 MeV for oxygen, and a set of lines for nitrogen due to neutron inelastic scattering (2.31 MeV, 5.11 MeV) and neutron capture (10.8 MeV). Thus, we have used the flux averaged over the detector cell (F4) tally to determine the energy distribution of γ-rays reaching the detector and the F8 pulse height tally modified with the GEB card to take into account the energy resolution of the LaBr_3_:Ce:Sr detector, pair production, and Doppler broadening effects. The energy resolution was included in the following full-width-at-half-maximum (FWHM) parametrization: $R\left(E_{\gamma }\right)=\frac{1}{E_{\gamma }}\left(a+b\sqrt{E_{\gamma }+cE_{\gamma }^{2}}\right)$, where $E_{\gamma }$ is the γ-ray energy (in MeV), with the parameter’s values amounting to: $a={2.0\cdot 10}^{-4}\;\mathrm{MeV},\;b={2.2\cdot 10}^{-2}{\;\mathrm{MeV}}^{-1/2},\;c=0.5\;{\mathrm{MeV}}^{-1}$ [24]. All the spectra were generated with a 10 keV bin size and an energy threshold of 100 keV. Each of the performed simulations was conducted for 10^8^ histories, which correspond to 1 s of the interrogation time. We have studied the performance of the integrated sensor in a function of the distance from the suspected object, its mass, and the depth at which it was buried in the ground.

## 3. Results

**Figure 5:**
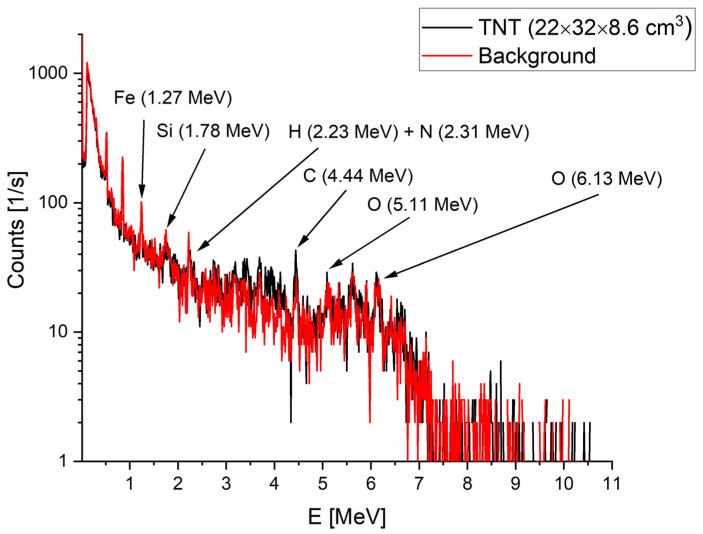
Simulated gamma ray energy deposition distribution simulated for background (red curve) and TNT mine (black). The sensor was placed 2 cm above the TNT.

An exemplary distribution of the simulated γ-ray energy depositions is shown in Figure 5 for a relatively large amount of TNT corresponding to the anti-tank MPP-B mine placed on the ground. In this case, the distance of the sensor from the charge is 2 cm. To estimate the environmental background, we also performed simulations without the presence of the mine (red curve in Figure 5). As expected, the background dominated the measurement and originated mostly from the ground (Si and O lines and the Compton scattering continuum originated from these γ-rays). The materials contained in the vehicle carrying the sensor also disturbed the lines of interest, especially hydrogen and carbon. The latter is, however, noticeably more abundant for the TNT in the 4.44 MeV region and for the escape peaks for this energy (3.93 and 3.42 MeV). The excess of signal over the estimated background is seen also for the oxygen line. Regarding nitrogen, the 2.31 MeV line is overwhelmed by hydrogen. Moreover, simulations revealed that the efficiency of the 2″ × 2″ LaBr_3_:Ce:Sr detector is too low to detect the 10.8 MeV line. A small nitrogen signal can be seen at 5.1 MeV, but this region is also populated by the double-escape peak of 6.13 MeV oxygen γ-rays.

To assess the performance of the sensor we have calculated integrals of H, C, N, and O peaks and compared their ratios for background and simulations with TNT. For all the results presented in this section the integrals were calculated within exactly the same bounds corresponding to the 3σ range around investigated lines. Since the MCNP simulation output is given together with relative uncertainty for each bin of the energy dependent F8, tally the standard deviation of every integral was computed conservatively as:

$$\sigma =\sqrt{\overset{m}{\underset{i=1}{\sum }}{\left(N_{i}\sigma_{i}\right)}^{2}}$$

where m is the number of bins of the F8 distribution contained in the integration range, $N_{i}$ denotes the content of the ith bin, and $\sigma_{i}$ is the corresponding relative uncertainty given by MCNP.

We have considered all the possible combinations of elemental ratios to determine the best observables for detection. Out of all the combinations, we have recognized the following ratios, which allowed us to distinguish the real signal from the background: C/O, C/H, C/N, and N/H. We have additionally checked if it is advantageous in the calculations to consider for oxygen and carbon, apart from the 4.44 MeV and 6.13 MeV lines, also the escape peaks mentioned before.. In the following subsections, we present the simulation results in terms of the listed elemental ratios. Their uncertainties are again calculated using the error propagation law using the variances of integrals for the two lines of interest. If the investigated ratio is expressed as $R=\frac{I_{1}}{I_{2}}$, and the corresponding standard deviation for the two integrated lines are $\sigma_{1}$ and $\sigma_{2}$, respectively, the uncertainty of R can be estimated using the following formula: $\sigma_{R}=\sqrt{{\left(\frac{\sigma_{1}}{I_{2}}\right)}^{2}+{\left(\frac{\sigma_{1}I_{1}}{I_{2}^{2}}\right)}^{2}}$.

### 3.1. Performance of the Detection as a Function of the Distance between the Sensor and the Tested Object

**Figure 6:**
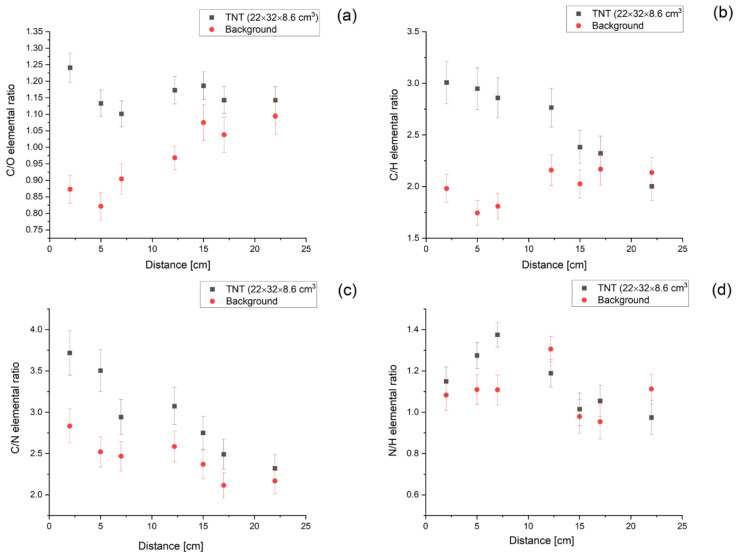
Elemental ratios of carbon and oxygen (**a**), carbon and hydrogen (**b**), carbon and nitrogen (**c**) and nitrogen and hydrogen (**d**), simulated for a sample of TNT (black) and background (red). In the calculations, we have taken into account the escape peaks for oxygen and carbon lines by adding their integrals to those for the original lines. These results correspond to the interrogation time of 1 s.

We have performed simulations for the 22 × 32 × 8.6 cm^3^ TNT mine changing the sensor distance from 2 to 22 cm. The results of our studies are presented for all the chosen rations in Figure 6. As one can see, all of them are consistent with the simulated background at a distance of about 20 cm. The simulations also show that the best performance can be obtained if one takes into account not only the standard oxygen and carbon lines but also the escape peaks (which we included by summing them to the 4.44 MeV and 6.13 MeV lines, respectively). Although the overall trends for background and signal are as expected, ratios in general decrease with the distance for TNT and increase for background; for some of the elements, the results change weakly with the distance. This may be result of the relatively large area of the assumed mine.

### 3.2. Feasibility Studies of the Anti-Tank Mines Detection in a Function of the Depth in the Soil

**Figure 7:**
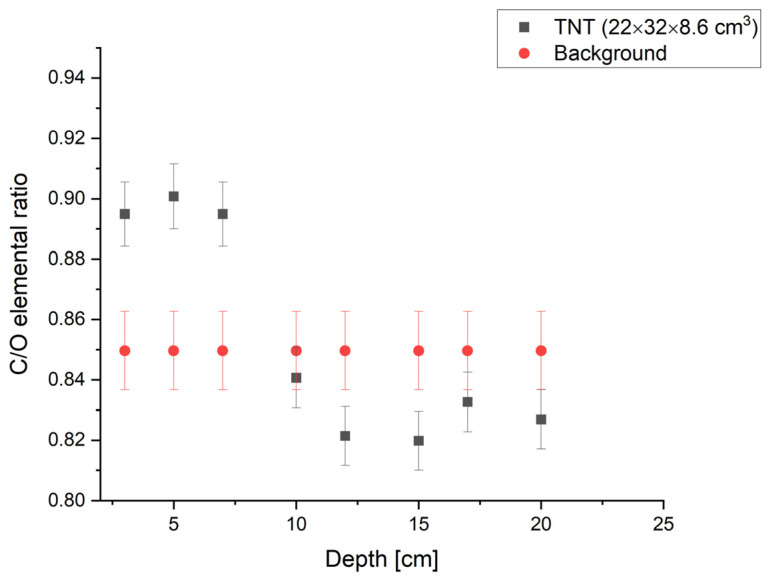
C/O elemental ratio as a function of the depth at which the TNT sample was buried for the detector positioned 2 cm above the ground.

This part of the simulations was performed for the same amount of explosives as presented in Section 3.1 and for the detector placed 2 cm above the ground. Simulations show that only the C/O ratio can be used to detect the buried explosive charge. Moreover, the interrogation time must be considerably higher to reduce the statistical fluctuations and uncertainties. In Figure 7, we present the dependence of the C/O ratio for the TNT sample buried at different depths. As one can see already at the depth of 10 cm with the simulated sensor design, we are not able to detect charges of about 10 kg at 10 cm depth.

### 3.3. Determination of the Minimal Detectable Mass of the Explosive Charges

**Figure 8:**
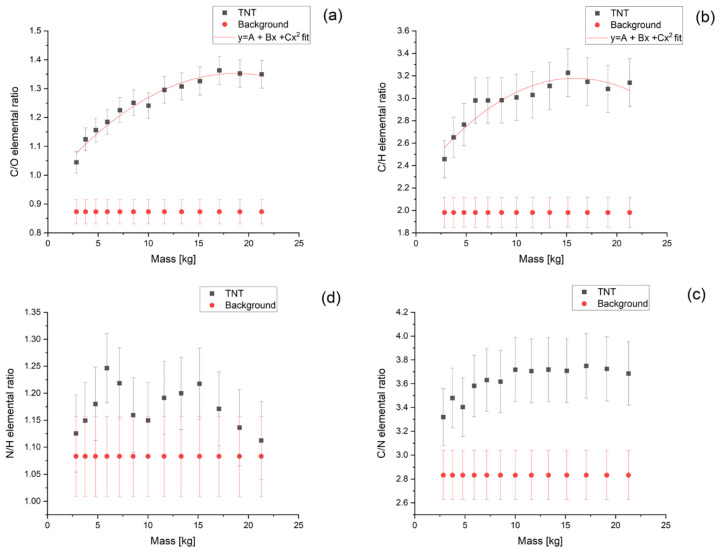
Elemental ratios of carbon and oxygen (**a**), carbon and hydrogen (**b**), carbon and nitrogen (**c**) and nitrogen and hydrogen (**d**), obtained for simulations performed for different masses of TNT with the γ-ray detector 2cm above the sample. The C/O and C/H ratios were fitted with a parabola function with parameter values equal to (**a**) A = 0.969 ± 0.021, B = 0.041 ± 0.005, C = 0.0011 ± 0.0002; (**b**) A = 2.262 ± 0.087, B = 0.116 ± 0.018, C = −0.0037 ± 0.0008.

The last item studied in the research described in this article was the determination of the minimal mass of the explosive substance which can be detected by our sensor. Here, we assumed the detector’s position 2 cm above the inspected object of 8.6 cm height. Its mass was changed from 2.8 kg to about 21 kg. The dependences of elemental ratios on the tested object mass obtained in the simulations are summarized in Figure 8.

Simulations show that in this case, the best discrimination between background and signal is given by the C/O and C/H ratios. We have fitted their dependence on the sample mass and used them to estimate the minimal amount of TNT which can be detected by the sensor. For both ratios, the mass of the charge can be as small as 100 g.

## 4. Discussion

We have performed a series of Monte Carlo simulations to assess the performance of a neutron-based sensor for the noninvasive detection of illicit substances integrated with a novel, remotely steered vehicle. It consists of the lightweight DT neutron generator by Thermo Fisher Scientific, P-320, and a γ quanta spectrometer made of 2″ × 2″ LaBr_3_:Ce:Sr crystal. This initial research constitutes the first step towards remote and fast detection of IEDs and terrorist threats using neutron beams. We have considered the performance of the sensor as a function of the distance to the tested object and the mass of the explosive charge. The simulations were also conducted for an amount of TNT corresponding to an anti-tank mine buried at different depths in the soil. The performance of the sensor was investigated in terms of elemental ratios for the C, N, O, and H lines simulated for explosives samples and background. Results of the simulations show that for the assumed sensor setup, successful detection of the TNT mine of about 10 kg can be achieved from a distance of up to 20 cm within 1 s of measurement considering the C/N, C/H, and C/O ratios. The latter two can be used to recognize a TNT charge down to about 100 g placed on the ground with the detector very close to the inspected object. This result points to the fast (within 1 s) and efficient detection of some of the anti-personnel mines and small IEDs. Much worse performance was observed for TNT charges buried in the ground. In this case, the interrogation time must be increased to 100 s and the maximum depth of the mine for which it can be recognized is less than 10 cm.

Results obtained with the performed simulations confirm somewhat expected facts—that the background induced by the neutron interactions with the soil and the vehicle materials are dominate the measured γ-ray spectra. Moreover, the dominating factor decreasing the performance of detection is the distance to the inspected object. Thus, the application of any method allowing for a decrease in the environmental background described in Section 1 would increase considerably the performance of detection. This will be the next step in the development of our sensor.

In the longer term, we plan also to supply the sensor based on neutron activation techniques with other devices like e.g., magnetometer, gravimeter, or precise positioning system. Both, the background reduction and decision processes will be supported by neural network-based algorithms applied at the level of data reconstruction and analysis.

## 5. Conclusions

In this paper, we have presented an integrated unmanned ground vehicle with a neutron-based sensor for the non-invasive detection of illicit substances. Currently, there are many applications of mobile robots that allow human explosives specialists to perform their tasks in safe conditions. Such remote applications require selective and precise detectors of explosives and other hazardous materials. This technology should operate in real-time mode, searching for items or resources in unconstrained and unknown environments. Performed simulations confirm successful detection of the typical TNT mine from distance of about 20 cm, which allows the use of remotely controlled platforms in real environments.

An advanced, modular, low-cost wheeled vehicle equipped with a neutron-based sensor using a properly sized manipulator arm was used for our research purpose. Dimensions of the platform as well as payload capacities were analyzed in terms of detector size, weight, and inspection capabilities. The platform has a control system designed for remote control with some autonomous functionality using various types of sensors and communication interfaces. Despite high-resolution cameras being used for teleoperation mode, additional perception sensors will support the operator for the successful navigation and localization of the robot in its workspace. The proposed architecture can be upgraded relatively easily by simply inserting or removing modules according to the previous experience from several projects carried out by the Military Institute of Armoured and Automotive Technology. Due to remote operation, it is necessary to ensure a stable wireless connection with the unmanned ground vehicles and to transfer a certain amount of data related to video streaming from cameras, as well as control signals, measurement results, etc. A radio system based on the mesh network has implemented a self-healing algorithm that automatically defines the best route to exchange the data. Each node in the network has two-way communication, which means it can receive and transmit information. It can easily extend the wireless operation range of mobile robots using path diversity in case some devices lose connection.

In relation to the observed influence of measurement background (due to operation environment as well as the mobile platform), additional acquired data (including orientation of generator and detector, and distance to the object) will be transferred to the computation unit to improve detection resolution by the developed algorithm. Such an approach will require additional research and investigation taking into consideration the hidden location of illicit materials in a real environment.

## Figures and Tables

**Table 1:** Main UGV parameters.

Parameter	Value
External dimensions	110 cm × 90 cm × 60 cm
Clearance	20 cm
Wheelbase	50 cm
Weight	100 kg
Drive	Direct current (DC) electric motors
Maximal speed	10 km/h
Operational time	5 h

## Data Availability

Publicly available datasets were generated and analyzed in this study. This data can be found here: https://ujchmura-my.sharepoint.com/:f:/g/personal/michal_silarski_uj_edu_pl/EiGatkEtMkhNtzZryA7g2m8BmfohqTHIxE4PegUcRegBXA?e=nHwVJ5 (accessed on 1 November 2022).
